# Hydrogels assembled from hybrid of whey protein amyloid fibrils and gliadin nanoparticles for curcumin loading: Microstructure, tunable viscoelasticity, and stability

**DOI:** 10.3389/fnut.2022.994740

**Published:** 2022-08-25

**Authors:** Yuqing Zhu, Yalan Han, Shengfeng Peng, Xing Chen, Youfa Xie, Ruihong Liang, Liqiang Zou

**Affiliations:** ^1^State Key Laboratory of Food Science and Technology, Nanchang University, Nanchang, China; ^2^Department of Food Science and Technology, Faculty of Science, National University of Singapore, Singapore, Singapore; ^3^Library of Nanchang University, Nanchang, China; ^4^School of Life Sciences, Nanchang University, Nanchang, China; ^5^Jiangzhong Pharmaceutical Co. Ltd., Nanchang, China

**Keywords:** whey protein isolate fibrils, gliadin nanoparticles, hydrogel, hybrids, viscoelasticity, curcumin, stability

## Abstract

Food grade hydrogel has become an ideal delivery system for bioactive substances and attracted wide attention. Hybrids of whey protein isolate amyloid fibrils (WPF) and gliadin nanoparticles (GNP) were able to assemble into WPF-GNP hydrogel at a low protein concentration of 2 wt%, among which WPF and GNP were fabricated from the hydrolysis of whey protein isolate under 85°C water bath (pH 2.0) and antisolvent precipitation, respectively. Atomic force microscope (AFM) images indicated that the ordered nanofibrillar network of WPF was formed at pH 2.0 with a thickness of about 10 nm. Cryo-SEM suggested that WPF-GNP hydrogel could arrest GNP within the fibrous reticular structure of the partially deformed WPF, while the hybrids of native whey protein isolate (WPI) and GNP (WPI-GNP hybrids) only led to protein aggregates. WPF-GNP hydrogel formed at pH 4.0 (85°C, 3 h, WPF:GNP = 4:1) possessed the largest elastic modulus (G’ = 419 Pa), which far exceeded the elastic modulus of the WPI-GNP hybrids (G’ = 16.3 Pa). The presence of NaCl could enhance the strength of WPF-GNP hydrogel and the largest value was achieved at 100 mM NaCl (∼10^5^ mPa) in the range of 0∼500 mM due to electrostatic screening. Moreover, WPF-GNP hydrogel showed a high encapsulation efficiency for curcumin, 89.76, 89.26, 89.02, 85.87, and 79.24% for pH 2.0, 3.0, 4.0, 5.0, and 6.0, respectively, which suggested that the formed hydrogel possess good potential as a delivery system. WPF-GNP hydrogel also exhibited a good protection effect on the photodegradation stability of the loaded curcumin with the retention of up to 75.18% after hydrogel was exposed to ultraviolet radiation for 7 days. These results suggested that the viscoelasticity of WPF-GNP hydrogel was tunable *via* pH-, ion-, or composition-adjustment and the hydrogel showed excellent protection on the thermal and photodegradation stability of curcumin.

## Introduction

Hydrogel, a soft material that often contains three-dimensional networks and large contents of aqueous fluid has been widely applicated in many areas, including biomedicine, material science, cosmetics, and food industry, in which they are often used as food additives (1), drug carriers (2), biosensors (3), biomaterials in tissue engineering (4), and 3D printing (5). Furthermore, hydrogels are also common delivery systems that can protect, deliver, and enhance the stability and bioavailability of drugs, antibodies, and bioactive ingredients (6–9) owning to their good biocompatibility and drug loading capability. In recent years, stimuli-responsive hydrogels, also called smart hydrogels, have attracted extensive attention due to their accurate response behavior to the variation of external processing conditions, such as pH values, ionic strength, temperature, UV irradiation by changing their conformation, solubility, and hydrophile-lipophile balance (10–12). Stimuli-responsive hydrogel could exhibit a sol-gel transition based on the changes in acid-labile bond, photocleavable moiety, or enzyme-substrate with a variety of physical, chemical, or biological stimuli, which can be used to modulate the mechanical or functional properties of the resulted hydrogels or achieve good control of local or duration of drug release. However, the application of stimuli-responsive hydrogels in the food industry is often limited by the raw materials and preparation processes which often involve organic reagents or sophisticated chemical reactions and may cause potential risk to the consumers. Herein, there is a broad space for the development of food-grade stimuli-responsive hydrogel based on natural biopolymers to enhance the digestion and absorption of bioactive substances by regulating the structure of carriers and controlling the release process, which would have huge potential development in the food industry.

For the past few years, there have been intensive research focused on the hydrogels assembled from the interactions of natural biological macromolecules (13–16). However, the formation of protein- or polysaccharide-based hydrogel requires a relatively high concentration (usually higher than 5% w/v) and the native gelatinous properties of the raw materials (17–19). Therefore, researchers have devoted a lot of efforts to preparing hydrogel using the hybrid interactions of composite ingredients, and large number of studies have reported the successful formation of hydrogel through the interactions between biopolymers (proteins and polysaccharides) and inorganic ions (Ca^2+^, poly-sulfobetaine, etc…) (20, 21), which can effectively enhance the gelation properties or functional properties of hydrogels. However, few studies have reported the fabrication of food-grade hydrogel through the structural design of proteins, and the possibility of fabricating hydrogel through designing and rebuilding the structures of original biopolymers to obtain ideal properties expecting to be further explored up to now.

Amyloid fibrils are formed based on the self-assembly of β-sheet of native proteins into twisted or helical ribbons and sequentially possess a high aspect ratio and mechanical strength, which endows them with resistance to digestive enzymes and stability under environmental stresses (22). Amyloid fibrils have attracted great interest in functional materials, which sever as aerogel, catalytic scaffold, and delivery systems in recent years (23–25). The helical ribbons structures of protein fibrils make them easier to bond with other substances to form cross-linked structures. Interestingly, fibril-nanoparticle hybrid hydrogels with tunable gelation behavior could be achieved through the intermolecular interactions between amyloid fibrils and nanoparticles (26, 27). These stimuli-responsive hydrogels also exhibited excellent functional properties and capacity in protecting, loading, and delivering microelements or bioactive ingredients (28, 29). These researches mainly focused on the interactions between fibrils and inorganic nanoparticles (Fe_3_O_4_, SiO_2_, etc…), as well as the application of building functional synthetic materials. Whether naturally sourced fibrils and nanoparticles can achieve similar effects remains unclear and the possibility of preparing hydrogel based on the hybrid of fibrils and nanoparticles is still waiting for exploration. Therefore, the efforts toward the fabrication of hydrogel based on different structures and conformations of biopolymers and modulation of the interactions between different components may provide a new sight in the design and utilization of functional hydrogel in the food area.

Whey protein isolate fibril (WPF) is a super-molecule derived from the self-assembly process of globular whey protein isolate (WPI) under acidic and heat treatment (30). Compared with native WPI, this fibrous shape is composed of larger contents of β-sheet and is highly ordered, leading to higher viscosity and gelation properties, which makes it easier to interact with other substances. A previous study has reported that the Fe_3_O_4_-modified β-lactoglobulin fibrils could easily form reversible hydrogels at low protein contents (2 wt%) under certain external stimuli (27), which suggested that the presence of nanoparticles could contribute to the interactions between protein molecules and lead to the formation of stimuli-responsive hydrogels. Our previous works have shown that gliadin could be self-assembled into nanoparticles under the changes of solubility in water and could be used to co-stabilize stimuli-responsive pickering emulsion gels with WPI through electrostatic, hydrophobic, and covalent interactions due to the sensitivity of gliadin nanoparticles (GNP) (31, 32). GNP, therefore, was selected as another component to prepare a fibril-nanoparticle stimuli-responsive hydrogel in this work, for its extensive cysteine residues which may be prone to interact with other molecules through disulfide bonds, which makes gliadin an ideal material for preparing protein-based hydrogel. Subsequently, curcumin was selected as a model drug to evaluate the potential of the formed hydrogel as a new delivery system. The effect of the ratio of GNP and WPF, pH values, ionic strength, and temperature on the fabrication and properties of hydrogel were characterized. Finally, the protective effect of hydrogel on the thermal stability and photodegradation of loaded curcumin was measured to evaluate the potential of the fibril-nanoparticle hybrid hydrogel as food-grade delivery system.

## Materials and methods

### Materials

Gliadin was extracted from commercial wheat proteins (≥80% purity, Xunxian Tianlong flour Co., Ltd) with a protein content of 92.6% according to our previous work (31). WPI powders were purchased from Hilmer (90% purity), Co., Ltd., United States. All other chemical reagents, such as HCl, NaOH, and ethanol were analytical grade and purchased from Xilong Scientific Co., Ltd (Lanzhou, China).

### Preparation of whey protein isolate fibril

Whey protein isolate powder was dissolved in deionized water under continuous stirring to obtain a 2% (w/v) WPI solution and then hydrated overnight at 4°C, and 0.01% (w/v) sodium azide was added to suppress microbial growth. The pH of the fully hydrated WPI was quickly adjusted to 2.0 with 6 M HCl and then subjected to heat in an 85°C water bath for 5 h with constant agitation. Whereafter, the stock solutions containing fibrils were cooled in an ice bath. Finally, the fibrils were collected for further experiments.

### Fabrication of gliadin nanoparticles

An aliquot of 2 g gliadin powder was dissolved in 100 mL 70% (v/v) ethanol in water solution under constant stirring overnight to ensure complete dissolution, followed by centrifuged (4,800 *g*) for 20 min to remove the insoluble substances. Then, the supernatant was collected and dropped into 250 mL 1% (v/v) acetic acid solution accompanied by stirring for 1 h. The ethanol in the gliadin solution was removed by vacuum-rotary evaporation at 45°C. Finally, the volume of the gliadin dispersion was adjusted to 200 mL and treated with a high-pressure microfluidizer at 120 MPa for two cycles to obtain GNP.

### Preparation of whey protein isolate fibril-gliadin nanoparticles hydrogel

Firstly, the pH values of WPI, WPF, and GNP solutions were adjusted to 2.0 with 6 M HCl for subsequent experiments. Secondly, the GNP solution was dropped into WPF or WPI solution under constant stirring and then incubated in an 85°C water bath for 3 h to form WPI-GNP hybrids and WPF-GNP hydrogel with a variety of volume ratios of these suspensions (WPF or WPI:GNP = 1:0, 1:1, 2:1, 3:1, 4:1, 0:1). Meanwhile, the effect of pH values (2.0–6.0) on the formation of WPI-GNP hybrids and WPF-GNP hydrogel was determined at a volume ratio of 4:1 (WPF or WPI:GNP) and 3 h heat treatment (85°C water bath). Native WPI-GNP hybrids were selected as a control group in the preparation of WPF-GNP hydrogel.

### Particle size distribution and zeta potential

The particle size distribution and zeta potential of WPI-GNP hybrids and WPF-GNP hydrogel with various ratios of WPF or WPI:GNP at pH 2.0–6.0 were determined with a dynamic light scattering instrument (Zetasizer Nano ZSP, Malvern Instruments Ltd, Worcestershire, United Kingdom). The dispersions were diluted 100 times with deionized water of corresponding pH values before being measured. The scattering angle of the instrument was fixed at 90° and the wavelengths of the lasers were 633 and 532 nm, respectively. Each measurement was conducted at least three times.

### Microstructures

The microstructures of WPF under different pH values were characterized using atomic force microscopy (AFM, Bruker, Germany). First, 2% (w/v) solutions were diluted 400 times to get a final concentration of 0.005% (w/v) fibril, sequentially 20 μL dispersion was dropped onto freshly prepared cleaved mica and equilibrate for 3 min. Then, the redundant samples were removed by rinsing with deionized water and dried under flow nitrogen. AFM tests were conducted with a Dimension edge (Bruker, Germany) using tapping mode at ambient temperature. The microstructures of WPF-GNP hydrogel and WPI-GNP hybrid (4:1, pH 4.0, heat for 3 h) were further detected by Cryo-scanning electron microscope (Cryo-SEM, SU8000, HITACHI); first, the samples were placed on a mental deck and then put into liquid nitrogen for a few seconds to solidify the samples. Second, the frozen samples were cut by a microtome (PP3010, Quorum Technologies Laughton, England) to obtain a flat surface and then do a gold sputtering. Third, the mental deck was transferred to a distillation device to sublimate the water (–95°C, 20 min). Finally, the microstructures of the samples were characterized.

### Mechanical properties

The rheological properties of WPF-GNP hydrogel and WPI-GNP hybrid with different ratios (WPI or WPF:GNP = 1:1, 2:1, 3:1, 4:1) and pH values (pH 2.0–6.0) after 3 h heat treatment were characterized by a Rotary Rheometer (MCR302, Anton Par, Germany) with a circular plate rotor (pp-50, 50 mm diameter), and the gap between the pp 50 and sample stage was set as 1 mm. About 2 mL of freshly prepared sample was evenly coated on the stage and equilibrium at 25°C for 5 min before measurements. First, the linear viscoelastic region (LVR) was tested under strain sweep mode with the strain range of 0.1% to 100% at the fixed frequency of 1 Hz. Then, oscillatory sweep was conducted at 1% strain with the frequency increased from 0.1 to 100 rad/s. Subsequently, the impact of NaCl concentration (0–500 mM) on WPF-GNP hydrogel (WPF:GNP = 4:1) at pH 4.0 was characterized, and then the impact of pH values (2.0–6.0) on WPF-GNP hydrogel (WPF:GNP = 4:1, NaCl = 100 mM) was tested using frequency sweep (1–100 rad/s).

### Encapsulation efficiency, thermal stability, and photodegradation of curcumin loaded in whey protein isolate fibril-gliadin nanoparticles hydrogel

First, 20 mg curcumin was dispersed in 2 mL pH 10.0 deionized water to obtain a 10 mg/mL stock solution. Curcumin was encapsulated in WPF using the pH-driven method, briefly, 0.25 mL curcumin stock solution was dropped slowly into pH 2.0 WPF dispersions under constant stirring, followed by 2 mL GNP added to the mixtures to form curcumin-loaded WPF-GNP hydrogel (WPF-GNP-C). The pH values of the hydrogels were adjusted to 2.0, 3.0, 4.0, 5.0, and 6.0, respectively. The encapsulation efficiency (EE) of curcumin was calculated as the following equation (33):


$$EE(\%)=\frac{\mathrm{curcumin}\mathrm{concentration}\mathrm{in}\mathrm{hydrogel}}{\begin{array}{c} \mathrm{curcumin}\mathrm{concentration}\mathrm{added}\mathrm{to}\mathrm{hydrogel}\;\mathrm{solution}\times 100\% \end{array}}$$

About 0.05 g curcumin was dissolved in 50 mL ethanol and then diluted four times to obtain 0.25 mg/mL curcumin solution and then used as a control group, the final curcumin concentration was 0.25 mg/mL. The freshly prepared WPF-GNP-C hydrogel was immediately transferred to a sealed glass bottle and then subjected to 85°C water bath for 180 min, and 1 mL WPF-GNP-C hydrogel was taken for testing the curcumin retention every 60 min. On the other hand, the freshly prepared WPF-GNP-C hydrogel with 3 h heat treatment (85°C) was placed in a 15-mm plastic petri dish under the UV-irradiation for 7 days and 1 mL WPF-GNP-C hydrogel was taken for testing the curcumin photodegradation every 1 day. Each measurement was conducted three times and the averages were recorded.

## Results and discussion

### Formation of whey protein isolate fibril-gliadin nanoparticles hydrogel

**FIGURE 1:**
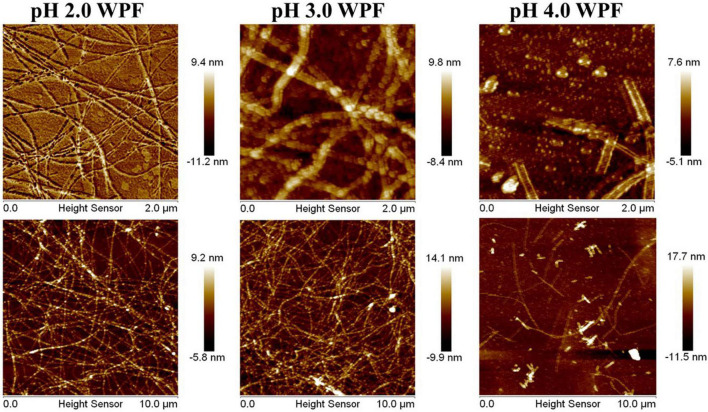
Atomic force microscope images of whey protein fibrils (WPF) at pH 2.0–4.0.

In brief, the microstructures of WPF at various pH values were observed through AFM, and the formation of semiflexible fibrous structures of WPI through thermal hydrolysis under acidic conditions was shown in Figure 1. The ordered nanofibrillar network could be easily observed at pH 2.0 and 3.0 with a thickness of about 10 nm. With the pH values increasing to 3.0 and 4.0, partial deformation and little aggregates could be seen in the horizon, which may be attributed to the protein aggregation near neutral pH conditions. Especially in pH 4.0, WPF exhibited an extensive degradation and the diameter of fibrils showed a significant increase with some little aggregation appearing. These results suggested that the formed fibrils were quite sensitive to pH values and possessed different micromorphologies in response to acidic conditions.

**FIGURE 2:**
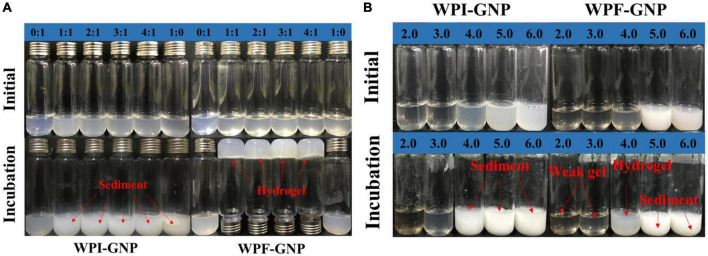
**(A)** Visual appearances of WPI-GNP hybrid and WPF-GNP hydrogel at various ratio and pH 4.0 before and after incubation at 85°C for 3 h; **(B)** The influence of pH values on the WPI-GNP hybrids (WPI: GNP = 4:1) and WPF-GNP hydrogel (WPF: GNP = 4:1) before and after incubation at 85°C for 3 h.

To investigate the gelation process of hydrogels, the effect of a series of protein volumes (WPI or WPF: GNP = 0:1. 1:1, 2:1. 3:1, 4:1, 1:0, pH 4.0) and pH values (2.0. 3.0, 4.0, 5.0, 6.0, WPI or WPF: GNP = 4:1) on the formation of hydrogels were studied, of which the hybrids of native WPI and GNP used as control. After being heated in an 85°C water bath for 3 h, there were intensive sediments in the bottom of the glass bottle of WPI-GNP hybrids (Figure 2A), and the content of the sediments increased with the increase of WPI concentration, which may be ascribed to the unfolding and aggregation of globular WPI molecules induced by thermal treatment (34). On the contrary, in the case of after WPI being converted to fibrils, there were upside-down hydrogels at the bottom of the bottle at the ratio of 1:1 to 4:1 (WPF:GNP), while the individual GNP or WPF solutions remain liquid-like. This phenomenon could be owing to the formed disulfide bonds and electrostatic interactions between GNP and WPF under thermal processing (32). Figure 2B shows that the complex of WPI and GNP were quite sensitive to the changes in pH values and prone to aggregate near their isoelectric point (pH 5.0–6.0), besides thermal treatment could accelerate this process. For the complex of WPF and GNP, the sediments immediately appeared at pH 5.0 and 6.0, and the aggregates tend to grow obviously after heat treatment. There were weak gels in the samples at pH 2.0 and 3.0 in the bottle of WPF and GNP, while larger gel strength of WPF-GNP hydrogel was present at pH 4.0 (Figures 2A,B). These results suggested that the formation process of hydrogel had strong pH-dependency and temperature-dependency, which may be attributed to the interactions of GNP and the partial deformed WPF.

**FIGURE 3:**
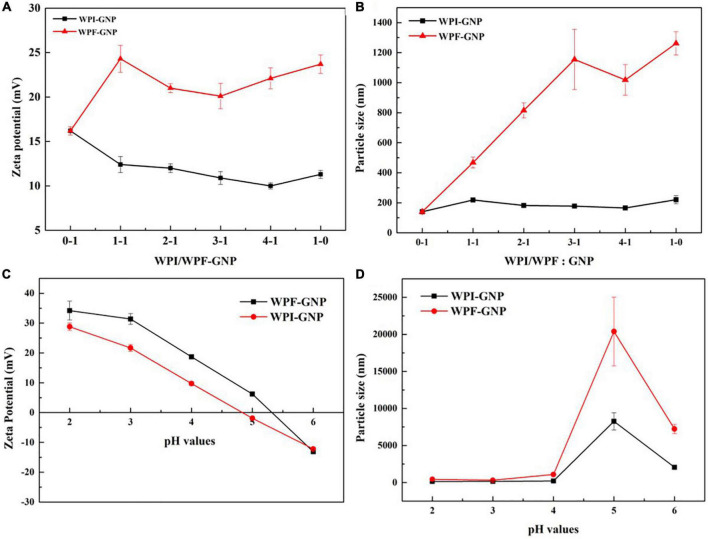
**(A,B)** Zeta potential and particle size distribution of WPI-GNP hybrids, WPF-GNP hydrogel at various ratio (pH 4.0); **(C,D)** Zeta potential and particle size distribution of WPI-GNP hybrids, WPF-GNP hydrogel at ratio of 4:1 with pH values ranging from 2.0 to 6.0.

The impact of protein ratio and pH values on the formation of hydrogels were further investigated by measuring the changes in particle size distribution and zeta potential of WPI-GNP hybrids and WPF-GNP hydrogel (Figure 3A). The zeta potential of native WPI or GNP was slightly larger than WPI-GNP complex, suggesting that there were interactions between WPI and GNP, which caused the changes in zeta potential. There was a slight decrease in the average zeta potential of WPI-GNP with the increase of WPI level, suggesting the electrostatic properties of the complex were mainly dominated by WPI. However, the magnitude of zeta potential of WPF-GNP hydrogel presented a noteworthy increase after the formation of protein fibrils and the absolute values tended to decrease first and then increase, and finally, the zeta potential of hydrogel was quite close to only WPF. This phenomenon illustrated that the thermal treatment of the WPI under acidic conditions could lead to the exposure of internal charged groups of WPI and the electric charges of WPF-GNP hydrogel had fibril concentration-dependency. Unlike the gentle change in zeta potential, the variation in the particle size distribution of hydrogels formed by WPF and GNP was quite significant, which may be attributed to the unfolding of globule protein molecules and the formation of filamentous structure (Figure 3B). Interestingly, there was a slight descend in the ratio of 4:1 (WPF:GNP), which may be attributed to the formation of structures that particles packaged in fibrils, leading to the curl of fibrils. The influence of pH values on the particle size distribution and zeta potential of hydrogel are shown in Figures 3C,D, the value of zeta potential of WPF-GNP and WPI-GNP decreased from 34.2 ± 3.15 mV and 28.8 ± 1.20 mV to –13.1 ± 0.436 mV and –12.2 ± 0.557 mV with the pH values increased from 2.0 to 6.0, with the isoelectric point turned out around pH 5.0. Consequently, there were extensive sediments at pH 5.0 and 6.0 in both the samples, and the changes in particle size distribution (Figure 3D) were also confirmed in this conclusion and is constant with Figure 2B, suggesting a total collapse of the hydrogel structures. Especially in pH 5.0, the sharp rise in particle size can be attributed to the decrease in electrostatic repulsion around the isoelectric points of the two protein molecules (5.0 and 6.8, respectively) (32).

**FIGURE 4:**
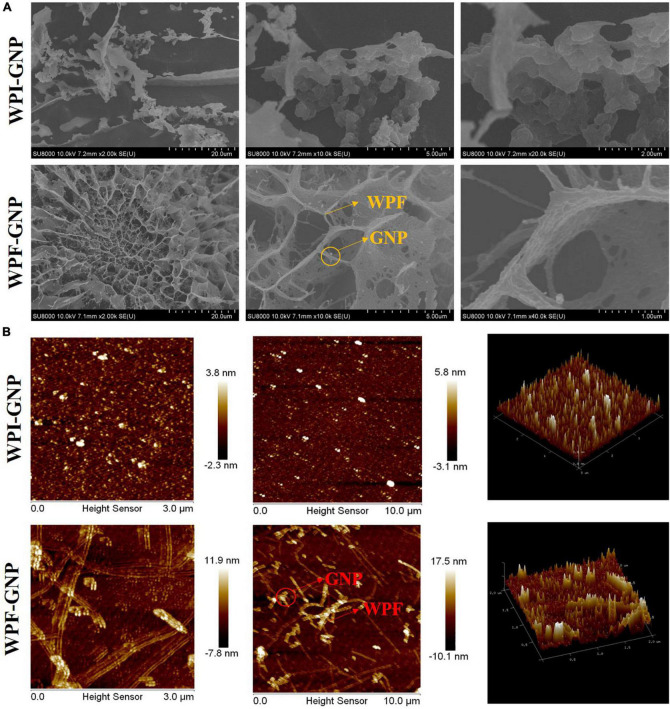
**(A)** Cryo-SEM images of WPI-GNP hybrids and WPF-GNP hydrogel at ratio of 4:1 and pH 4.0; **(B)** AFM images of WPI-GNP hybrids and WPF-GNP hydrogel at ratio of 4:1 and pH 4.0.

The microstructures with different magnifications of the WPI-GNP hybrid and WPF-GNP hydrogel (4:1, pH 4.0) formed after 3 h heat treatment were characterized using cryo-SEM and AFM and is shown in Figure 4. The WPI-GNP hybrid presents an agglomerative behavior, suggesting the formation of aggregates or sediments between WPI and GNP under heating conditions, which was consistent with the macroscopical image (Figure 2A). From the three-dimensional image (Figure 4B), a few protein aggregates can be seen in the horizon and the diameter of fibrils broaden while the length shortens. After the addition of GNP, a spherical complex of proteins uniformly filled in the field of view, and several larger aggregates formed due to the interaction of GNP and WPI. As for WPF-GNP hydrogel, a fibrous network structure can be observed and some particles can be seen captured in the scaffolding of WPF (Figure 4A). There was also an obvious structure of the nanoparticle-filled-in-fibril network in the two-dimensional images (Figure 4B), which suggested that the presence of GNP could bond with fibrils and then promote the formation of hydrogel.

### Characterization of whey protein isolate fibril-gliadin nanoparticles hydrogel

#### Rheological properties of whey protein isolate fibril-gliadin nanoparticles hydrogel

**FIGURE 5:**
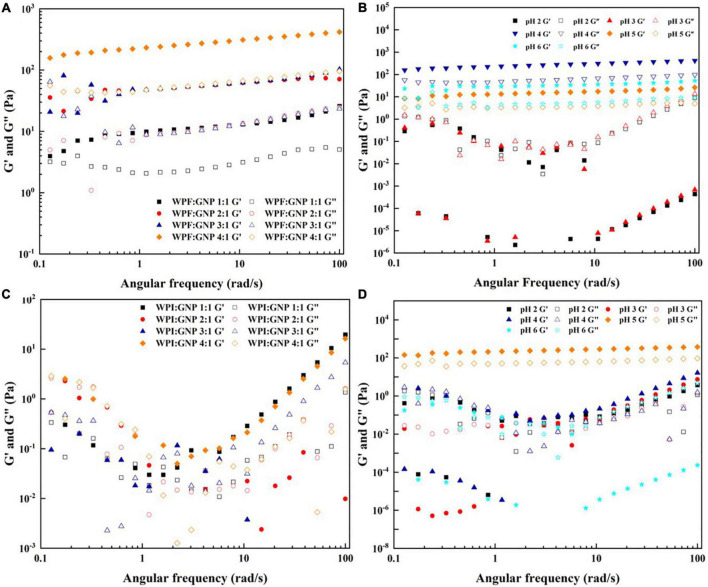
Frequency sweep of WPF-GNP hydrogel at various protein ratio **(A)** and pH values **(B)**; Frequency sweep of WPI-GNP hybrids at various protein ratio **(C)** and pH values **(D)**.

The pH and heat conditions are important factors that affect the structural properties of the formed WPF-GNP hydrogels and their application in the food industry, thus the impact of pH values and heating process on the rheological properties of WPF-GNP hydrogels were evaluated using rotary rheology. First, the influence of the ratio of WPI or WPF to GNP was determined by monitoring the changes of elastic modulus (G′) and loss modulus (G″). Figure 5A suggest that the value of G′ increased with the increasing of contents of WPF and the maximum figure for G′ showed at the ratio of 4:1 (WPF:GNP), which indicated that the hydrogel formed at 4:1 under 3 h heat treatment possessed the largest strength. Heating could promote the unfolding of globular proteins and the exposure of internal hydrophobic sulfhydryl groups, leading to the formation of disulfide bonds and hydrophobic interactions between protein molecules, which subsequently induced the partial aggregation of protein fibrils and reinforced the gel strength of hydrogels (32). Figure 5B shows that G′ of WPF-GNP hydrogels at pH 4.0 to 6.0 exhibited a steady tendency while the G′ of WPF-GNP hydrogels at pH 520 and 3.0 exhibited a chaos variation, which may be attributed to the aggregates of sediments formed around the isoelectric point of whey protein isolate. When it comes to the changes in the WPI-GNP hybrid, the figures for G′ and G″ were quite close to each other and irregular (Figure 5C), indicating that there were almost no obvious gel structures in all the systems, which can be attributed to the aggregation and structural collapse of globular protein molecules and under heat treatment. Similar to WPF-GNP hydrogel, pH values were also an important factor that affect the rheological properties of the WPI-GNP hybrid (Figure 5D), but interestingly, the WPI-GNP hybrid at pH 5.0 possessed the largest elastic modulus, which may be explained by the formation of large deposits of WPI around its isoelectric point. These results suggested that the interactions between nanoparticles and protein fibrils could be remarkably strengthened by the ratio of proteins and pH environments. All in all, pH conditions and protein ratio are useful approaches to preparing and adjusting the formation and characteristics of nanoparticle-fibril hydrogels.

**FIGURE 6:**
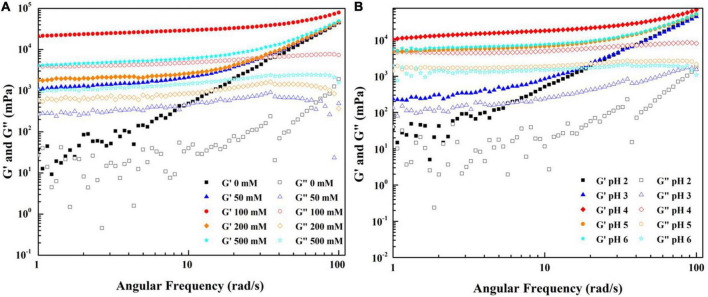
Frequency sweep of WPF-GNP hydrogel (WPF: GNP = 4:1) at various NaCl concentration (0–500 mM) **(A)** and pH values (2.0–6.0, NaCl = 100 mM) **(B)**.

The impacts of ionic strength and pH conditions combined without the heating process on the native WPF-GNP hydrogels are present in Figure 6. In general, the addition of NaCl could improve the gel strength of WPF-GNP hydrogel (Figure 6A). For the original hydrogel formed by GNP and WPF without heat treatment (NaCl = 0 mM), the dynamic frequency profile indicated that the values of G′ and G″ were quite disordered at a low-frequency range (1–10 Hz) but G′ gradually exceeded G″ at the following testing frequency range (Figure 6A), suggesting a weak gel structure in the mixture of GNP and WPF. In the presence of NaCl, the magnitude of elastic modulus of WPF-GNP increased with the growth of NaCl concentration, except for hydrogel containing 100 mM NaCl (Figure 6A), which possessed the highest elastic modulus (G′). This phenomenon may be ascribed to the electrostatic shielding of the salty ions, which weakened the electrostatic repulsion of the system and thus the interactions between protein molecules would be reinforced, cold-set WPF-GNP hydrogels with 100 mM NaCl can be macroscopically inverted and still stayed in the bottom of the tubes (data not show). However, the figures for G′ at 200 mM and 500 mM were smaller which may be owing to the aggregation formed between GNP and WPF. Our previous study suggested that the net charge of WPI and GNP decreased at 200 and 500 mM NaCl (32), leading to the decline of the interactions and an increase in the extent of aggregation in the WPF-GNP hydrogel system. In the presence of NaCl, the influence of pH values on the gelation properties was investigated (Figure 6B). The WPF-GNP hydrogel exhibited the largest elastic modulus at pH 4.0, followed by hydrogels at pH 5.0 and 6.0, while the elastic moduli at pH 2.0 and 3.0 were relatively smaller and showed a liquid-like state, which suggested that pH conditions is also a vital factor during the formation of hydrogel and could regulate the gel strength of hydrogel through altering the electrostatic interactions between protein molecules. Previous research also suggested that the variation of pH values could induce changes in protein molecular conformation and lead to changes in the type of interaction forces, such as noncovalent interaction or covalent interactions (35).

### Encapsulation efficiency, heat stability, and photodegradation stability of curcumin loaded in whey protein isolate fibril-gliadin nanoparticles hydrogel

**FIGURE 7:**
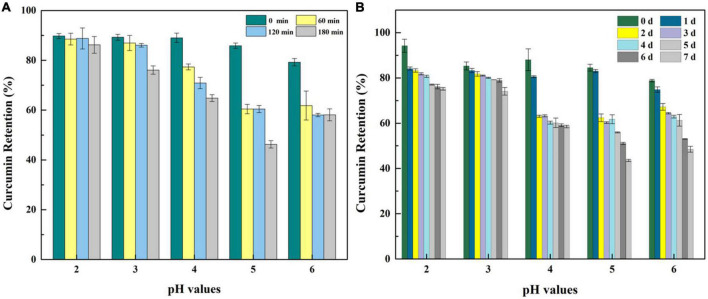
Curcumin retention of WPF-GNP-C hydrogel (4:1) after heated at 85°C for 3 h **(A)** and after ultraviolet radiation for 7 days **(B)** at various pH values in the absence of NaCl.

To investigate and evaluate the performance of hydrogels serving as a delivery system, curcumin was selected as a model bioactive ingredient to conduct further experiments. There were no significant differences in the visual appearance, colloidal properties, and microstructures of the hydrogel-loading curcumin except the gel strength which was slightly weakened at the ratio 1:1 of WPF to GNP (Supplementary Figures 1–3), which indicated that the addition of curcumin caused few effects on the characteristic of the hydrogel. First, the encapsulation capacity of hydrogel was characterized by the index of encapsulation efficiency (EE). As suggested in Figure 7A, WPF-GNP hydrogel showed a high EE for curcumin at all tested pH values, 89.76, 89.26, 89.02, 85.87 and 79.24%, for pH 2.0, 3.0, 4.0, 5.0, and 6.0, respectively, which suggested that the formed hydrogel possess good potential as a delivery system. The EE of the hydrogel at pH 5.0 and 6.0 was lower than other groups, which may be attributed to the sediments formed at pH 5.0 and 6.0 (around the isoelectric point of WPI and GNP), and the structure of hydrogel was partially destroyed, resulting in the decrease of EE. Moreover, the stability of the delivery system to various environmental stresses was also an important index to evaluate the quality of delivery systems; herein, heat and UV were selected as two representative environmental stresses to conduct further experiments. The heat stability of curcumin at various pH values was explored and the curcumin retention is shown in Figure 7A. There were slight losses of curcumin during the encapsulation process on WPF-GNP hydrogel, especially for hydrogel at pH 5.0, which may be attributed to the partial degradation of curcumin under rapid changes of acid-base environments. After being exposed to an 85°C water bath for 180 min, the curcumin retention in WPF-GNP hydrogel nearly remain at the original level at pH 2.0, while the degradation amount reached a minimum value (54 ± 1.45%) at pH 5.0 which can be due to the formation of aggregates or sediments in the hydrogel, leading to the reduction in loading capacity of hydrogel, hence the curcumin degradation after exposure to environmental stress (e.g., oxygen). With the increase in pH conditions, curcumin retention declined to 76.09 ± 1.65% at pH 3.0, 64.82 ± 1.40% at pH 4.0, and 58.11 ± 2.41% at pH 6.0 after heating at 85°C for 180 min, respectively, suggesting that the protective capability of WPF-GNP hydrogel on curcumin may decrease with the increasing in pH values, which may be ascribed to the gel-like structures formed at higher pH values that prevent curcumin from binding to the protein molecules. Compared to the control groups, WPF-GNP hydrogel showed obvious better protective effects on curcumin at most pH values except for pH 5.0 and 6.0, which may be attributed to the partial aggregation of hydrogel, thus hindering the encapsulation process of curcumin. Chen et al. reported a similar result of curcumin loaded in soy soluble polysaccharide nanoparticles that the retention of curcumin could reach a figure of nearly 80% after heating at 80°C for 2.5 h (pH 4.0), while the degraded ratio of free curcumin exceeded 30% (36). Previous studies also suggested that the phenolic hydroxyl groups of curcumin were easily ionized under acidic conditions, which avoid being oxidized; while in neutral conditions, the H^+^ of phenolic hydroxyl groups tend to lose and leaves curcumin in an unstable status, resulting in the oxidation of curcumin (37). Furthermore, the aqueous solubility of curcumin could be enhanced through complexation with whey protein fibrils in acidic conditions (38).

The photodegradation stability of curcumin loaded on WPF-GNP hydrogel at pH 2.0 to 6.0 for 7 days is present in Figure 7B. After exposure to ultraviolet radiation for 7 days, the amount of curcumin gradually decreased with the extent of exposure time and curcumin retention in different hydrogels were vastly different; WPF-GNP hydrogel exhibited better protection on curcumin under lower pH values and the retention could reach 75.18% at pH 2.0 and 74.10% at pH 3.0, which may due to the incorporation of curcumin into protein molecules. Hu et al. reported that hydrophobic polyphenols could bind to protein fibers and induce the formation of hydrogels through self-assembly of hybrid super-molecules (29). With the pH increased to 4.0, the degradation rate and amount of curcumin increased, which may be due to the deformation of WPF and subsequently the increased gel strength of hydrogels inhibiting the binding process of curcumin to protein; therefore, curcumin tends to disperse in the aqueous solution and hydrogels could not screen the effect of the ultraviolet light. When it comes to pH 5.0 and 6.0, the curcumin retention declined to 43.52 and 48.50%, respectively, and nearly half of the curcumin degraded after 7 days of UV irradiation, suggesting that the aggregates and sediments of hydrogels reduced its protective effect on curcumin, for curcumin could not bind to the fibrils tightly but only disperse in the mixture. Iurciuc et al. reported that the degradation of free curcumin exposed for 7 days to natural light and air reached a figure of 30% and the degradation half-time (t_1/2_) of free curcumin was 6.8 ± 0.3 h at pH 3.0 and 6.2 ± 0.3 h at pH 6.8 (39). A previous study reported that an increased gel strength of the delivery system usually means better protection from bioactive substances (40), which was opposite to our results. We speculated that the regular fibrous structure at lower pH values could encapsulate curcumin more tightly and shield it from external stimuli, while the partially deformed fiber structures at higher pH values would increase the exposure of curcumin to UV irradiation. These results indicated that the microstructures of WPF-GNP hydrogel could be effectively modulated by adjusting pH values, and this pH-responsive property of hydrogels could effectively regulate the photodegradation stability of curcumin.

## Conclusion

In this work, fibril-nanoparticle hybrid hydrogels were successfully prepared through the interactions of GNP and WPF at a low protein concentration of 2 wt%; the gel strength and fluid nature of which could be easily modulated by altering pH, ionic strength, or composition. Compared to native globular proteins, amyloid fibrils generated from WPI were easier to bind and capture GNP to form a gel network with improved strength. The largest gel strength was achieved at the WPF:GNP ratio of 4:1 and pH 4.0 after 3 h heat treatment, indicating that the partial deformation of WPF is more apt to bind with nanoparticles. The introduction of NaCl could further enhance the strength of WPF-GNP hydrogel, and the highest strength was reached in the presence of 100 mM NaCl. Moreover, the WPF-GNP hydrogel could effectively load and protect curcumin from thermal degradation and photodegradation. In particular, a higher strength of hydrogels did not result in higher resistance stability of incorporated curcumin to the environmental stresses, which may be ascribed to polyphenols that were more readily interacted with amyloid fibers in lower acidic media and curcumin was prone to degrade under higher pH conditions. These findings may provide a new insight in developing food hydrogels with tunable characteristics through the interactions between fibril and nanoparticles to enlarge the application of hydrogels in food ingredients or functional foods.

## Data availability statement

The original contributions presented in this study are included in the article/Supplementary material, further inquiries can be directed to the corresponding authors.

## Author contributions

YZ: conceptualization, methodology, investigation, and writing—original draft. YH: writing—review and editing. SP: formal analysis. XC and YX: writing—review and editing and visualization. RL: writing—review and editing. LZ: validation, methodology, and investigation. All authors contributed to the article and approved the submitted version.
