# Supplementary figures and images for: Hydrogels assembled from hybrid of whey protein amyloid fibrils and gliadin nanoparticles for curcumin loading: Microstructure, tunable viscoelasticity, and stability
Source: Front Nutr. 2022 Aug 25;9:994740. doi: 10.3389/fnut.2022.994740 (PMC9462383; doi:10.3389/fnut.2022.994740)

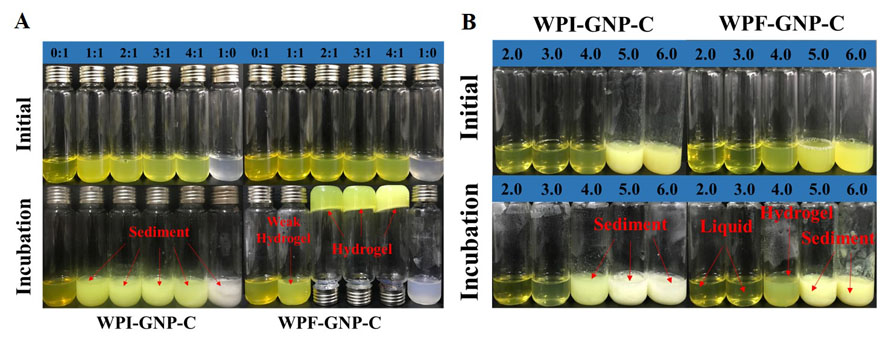

Supplement: Supplementary file 1 [file Image_1.JPEG]

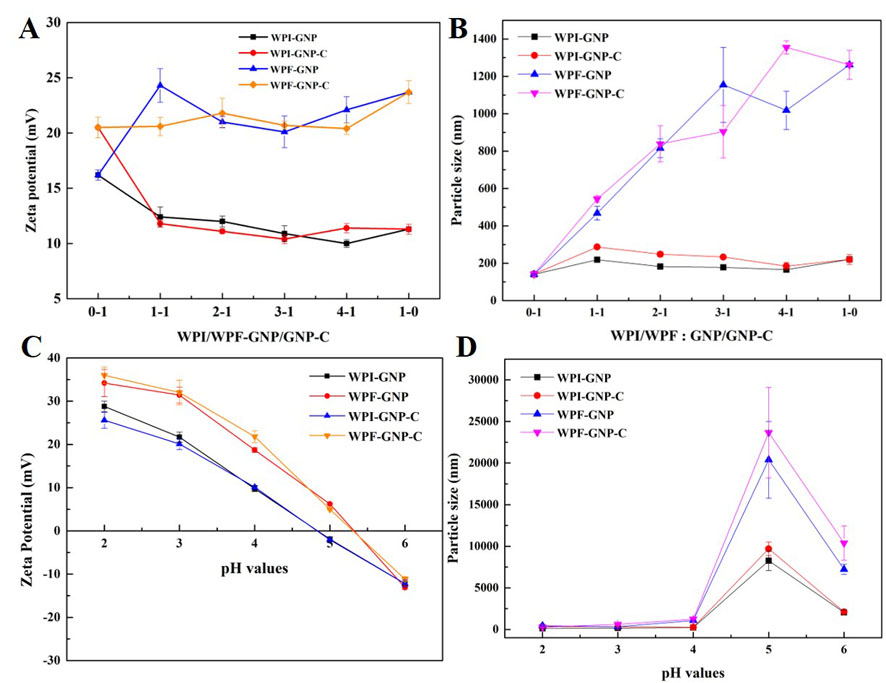

Supplement: Supplementary file 2 [file Image_2.JPEG]

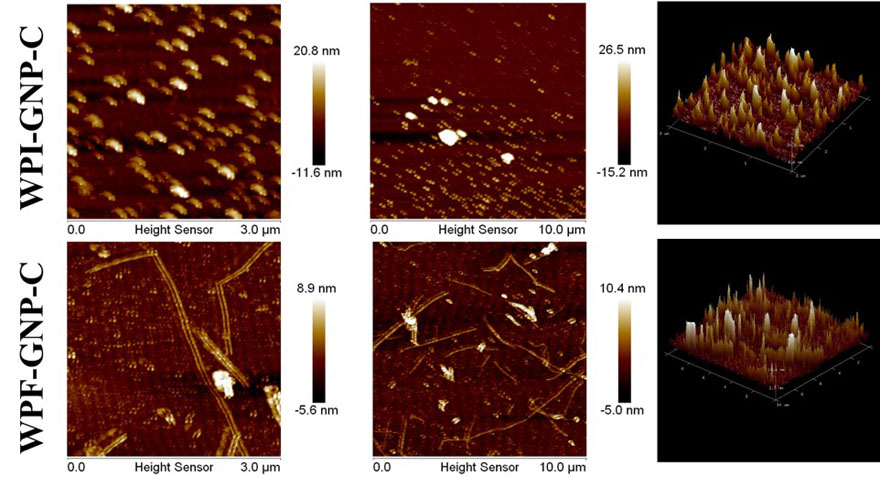

Supplement: Supplementary file 3 [file Image_3.JPEG]
